# Botanical Origin Differentiation of Malaysian Stingless Bee Honey Produced by *Heterotrigona itama* and *Geniotrigona thoracica* Using Chemometrics

**DOI:** 10.3390/molecules26247628

**Published:** 2021-12-16

**Authors:** Wen-Jie Ng, Nam-Weng Sit, Peter Aun-Chuan Ooi, Kah-Yaw Ee, Tuck-Meng Lim

**Affiliations:** Faculty of Science, Universiti Tunku Abdul Rahman (UTAR), Kampar 31900, Malaysia; sitnw@utar.edu.my (N.-W.S.); peteracooi@gmail.com (P.A.-C.O.); eeky@utar.edu.my (K.-Y.E.); ltmeng@utar.edu.my (T.-M.L.)

**Keywords:** *kelulut* honey, honeydew honey, blossom honey, NMR profiling, physicochemical, functional foods, antioxidant, honey differentiation

## Abstract

Stingless bee honey, specifically honeydew honey, is generally valued for its better health benefits than those of most blossom types. However, scientific studies about the differentiation of stingless bee honey based on honeydew and blossom origins are very limited. In this study, ^13^C NMR spectroscopy was employed to quantify the seven major sugar tautomers in stingless bee honey samples, and the major sugar compositions of both honeydew and blossom types were found not significantly different. However, several physicochemical properties of honeydew honey including moisture content, free acidity, electrical conductivity, ash content, acetic acid, diastase, hydrogen peroxide, and mineral elements levels were significantly higher; while total soluble solid, proline, and hydroxymethylfurfural were significantly lower than blossom honey. Greater antioxidant capacity in honeydew honey was proven with higher total phenolic compounds, ABTS, DPPH, superoxide radical scavenging activities, peroxyl radical inhibition, iron chelation, and ferric reducing power. Using principal component analysis (PCA), two clusters of stingless bee honey from the honeydew and blossom origin were observed. PCA also revealed that the differentiation between honeydew and blossom origin of stingless bee honey is possible with certain physicochemical and antioxidant parameters. The combination of NMR spectroscopy and chemometrics are suggested to be useful to determine the authenticity and botanical origin of stingless bee honey.

## 1. Introduction

Stingless bee is the largest group of eusocial bees on Earth with more than 500 described species. It is distributed in tropical and subtropical regions including tropical America, Africa, Australia, and Southeast Asia, where it is 50 times greater in number than honey bees [1]. Less attention and study have been focused on these bees and related products presumably because they are not found in North America and European regions [2].

The production of honey begins with nectar collection by bees from plants, whereby the bees transform and process the nectar with specific substances of their own. Instead of hexagonal-shaped combs, stingless bees store their nectar in small resin pots. To date, 32 stingless bee species have been documented in Malaysia [3], and *Heterotrigona itama* and *Geniotrigona thoracica* are the most common domesticated stingless bees for meliponiculture purpose. Recently, stingless bee honey, which is locally known as *kelulut* honey, is receiving more attention and demand from the public due to its distinctive sensory characteristics and health-promoting properties. Other than floral nectar, bees also harvest honeydew honey from plant secretions. Honeydew honey is highly valued because it is considered to possess more health benefits than floral or blossom honey [4]. Several studies also demonstrated that the antibacterial and antioxidant activities of honeydew honey are superior to those of blossom honey [5].

Although the quality parameters for honey have been defined by Codex Alimentarius Commission [6] and Council Directive of the European Union [7], they are not applicable for honey originating from tropical and subtropical regions, especially stingless bee honey [8]. Despite a specification standard for stingless bee honey formulated by the Department of Standards Malaysia [9] to regulate the quality of locally produced stingless bee honey, the quality requirements do not include differentiation of honeydew origin from blossom origin honeys. Furthermore, scientific studies about the differentiation of stingless bee honey based on honeydew and blossom origins are very limited. Hence, it is crucial to differentiate stingless bee honey to ensure authenticity and avoid adulteration. A systematic and comprehensive research that can profile the physicochemical properties and bioactivities of stingless bee honey is needed.

Thus, the main interest of this study was to differentiate the botanical origins of stingless bee honey using chemometric analysis. Such analysis is not only crucial for the characterization of stingless bee honey using physicochemical characteristics and antioxidant capacity but also important to ensure the quality of local stingless bee honey products for both domestic consumption and exports. 

## 2. Results

### 2.1. Physicochemical Properties

#### 2.1.1. Major Sugar Composition

^13^C NMR chemical shifts of the seven tautomers relative to 1,4-dioxane are reported in Supplementary Material Table S1. Tables S2 and S3 show the ^13^C NMR chemical shifts of the isoglucose and artificial honey, respectively. The comparison of actual concentration and ^13^C NMR determined concentration of sugars and tautomers is also shown in the same tables. The ^13^C NMR spectra of model compounds (Figure S1a–c), isoglucose (Figure S2), and artificial honey (Figure S3) are also provided as Supplementary Material. No significant changes in chemical shifts for each tautomer in isoglucose and artificial honey relative to the individual model compounds, indicating that the ^13^C chemical shifts of a sugar tautomer are not influenced by the presence of other sugars in a solution including honey [10,11]. The assignment of all carbon signals for honeydew and blossom honey (Figure S4) is based on the assigned ^13^C NMR spectra of the model compounds, isoglucose, and artificial honey. Table 1 summarizes the average concentration of each tautomer in honeydew and blossom honey. The content of glucose and fructose was not significantly different between honeydew honey and blossom honey. The content of sucrose in honeydew honey was too low to be integrated by ^13^C NMR spectroscopy.

#### 2.1.2. Maturity of Honey

As shown in Table 2, although the color intensity of honeydew honey with an average value of 173.75 mAU was darker than blossom honey (146.67 mAU), the difference was not significant. The moisture content in stingless bee honey samples ranged from 22.50% to 28.00%, with significant higher moisture content in honeydew honey (27.03%) than blossom honey (25.67%). The water activity in both honeydew honey (0.63) and blossom honey (0.62) were rather similar and slightly higher than 0.6. Contrary to moisture content, the total soluble solid in honeydew honey (72.97 °Brix) was detected to be significantly lower than blossom honey (74.32 °Brix). Proline content in honeydew honey (522.38 mg/kg) was found to be significantly lower than blossom honey (563.11 mg/kg).

#### 2.1.3. Purity of Honey

In Table 3, the values of both electrical conductivity and ash content showed great variability among botanical origins whereby values found in honeydew honey (0.45 mS/cm; 0.14%, g/100 g) were significantly higher than blossom honey (0.36 mS/cm; 0.04%, g/100 g). The diastase level in stingless bee honey samples reported in this study ranged from 1.83 to 3.04 Schade units. The hydrogen peroxide level of stingless bee honey samples in this study ranged from 111.07 to 192.82 µmol/L with a significantly higher value detected in honeydew honey (183.51 µmol/L) than blossom honey (137.61 µmol/L).

#### 2.1.4. Deterioration State of Honey

As shown in Table 4, although the pH values of honeydew honey and blossom honey were not to be significantly different, the free acidity value of honeydew honey (90.96 meq/kg) was found to the significantly higher than blossom honey (76.64 meq/kg). Although higher levels of organic acids were determined in honeydew honey, only the acetic acid level of honeydew honey (0.10 g/kg) was significantly higher than blossom honey (0.07 g/kg). The HMF content of both honeydew and blossom honeys were below 20 mg/kg.

#### 2.1.5. Mineral Profile

As displayed in Table 5, significantly higher levels of sodium (Na), potassium (K), magnesium (Mg), calcium (Ca), iron (Fe), and zinc (Zn) were measured in honeydew honey. However, the quantity of copper (Cu) and aluminum (Al) was too little to be quantified in both honey types. A significantly higher total content of mineral elements was determined in honeydew honey (732.91 mg/kg) than blossom honey (573.86 mg/kg).

### 2.2. Antioxidant Properties

Table 6 shows honeydew honey possessed greater antioxidant capacities than blossom honey. Other than significantly higher total phenolic content (104.09 mg GAE/kg), scavenging activities against ABTS, DPPH and superoxide radicals (63.15%, 35.65%, and 76.10%), peroxyl radical inhibition (5.71 µmol TE/g), iron chelation (18.94%), and ferric reducing power (3.18 mmol Fe(II)/kg) were all significantly higher than blossom honey.

### 2.3. Chemometric Analysis

**Figure 1:**
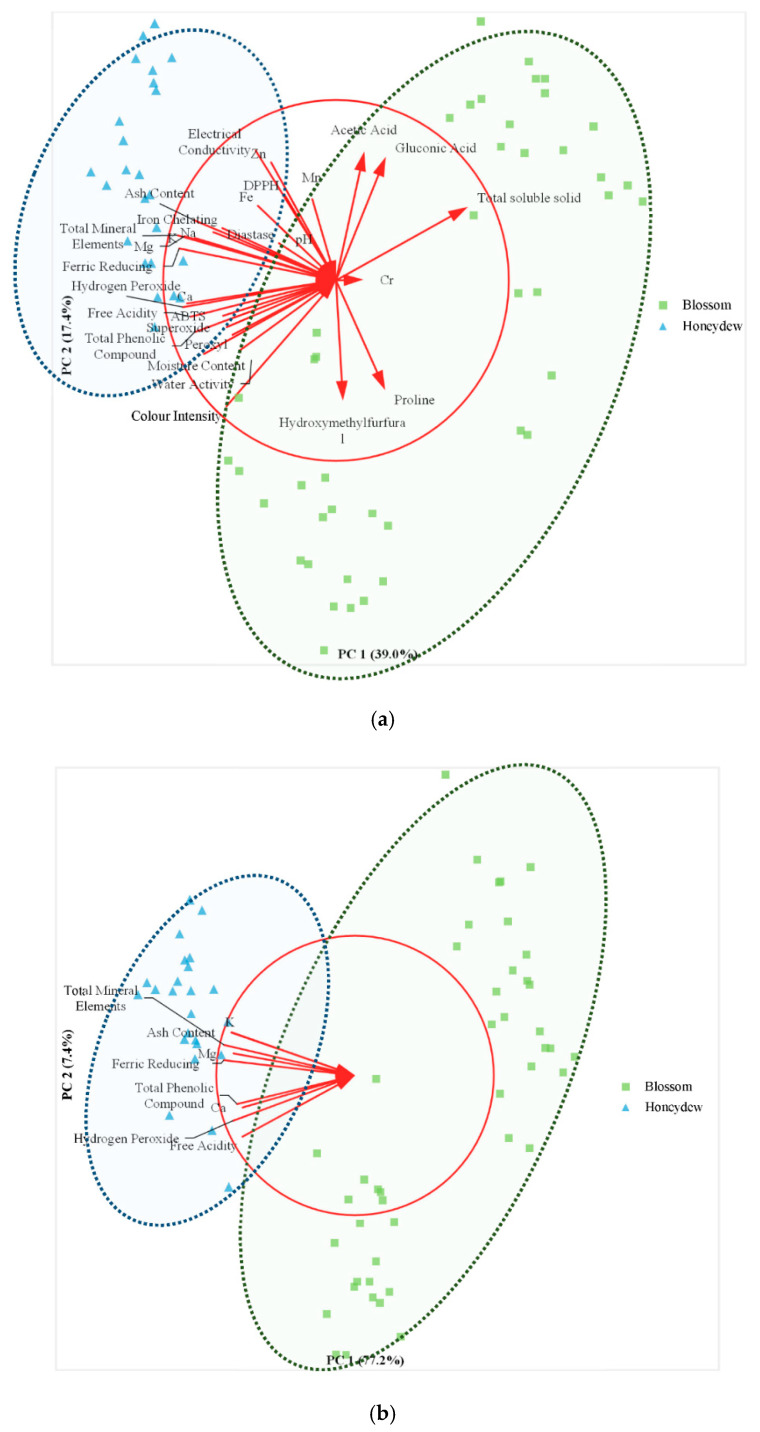
(**a**) Plot of principal component loadings stingless bee blossom and honeydew honey samples and the descriptors including physicochemical and antioxidant properties. (**b**) Plot of principal component loadings stingless bee blossom and honeydew honey samples and the descriptors including physicochemical and antioxidant properties.

As shown in Table 7, both first principal component (PC1) and second principal component (PC2) represented 56.40% of the variance. According to correlation coefficient, the parameters that most associated with PC1 were ash content (−0.822), hydrogen peroxide (−0.886), free acidity (−0.842), total mineral elements (−0.916), K (−0.817), Mg (−0.876), Ca (−0.863), total phenolic compounds (−0.817), and ferric reducing power (−0.907). Considering only these parameters, another PCA was generated with 84.60% of the total data variance. The correlation coefficients of these parameters were ash content (−0.891), hydrogen peroxide (−0.878), free acidity (−0.811), total mineral elements (−0.945), K (−0.892), Mg (−0.876), Ca (−0.810), total phenolic compounds (−0.851), and ferric reducing power (−0.945). This analysis was able to differentiate all the analyzed honey samples into two clusters based on honeydew and blossom origins (Figure 1).

## 3. Discussion

^1^H NMR spectroscopy is commonly used in food analysis; however, highly complex ^1^H NMR spectra can complicate the interpretation and profiling of food metabolites including honey [10]. Such an issue can be overcome by ^13^C NMR spectroscopy which has been largely employed for structural studies. The ^13^C NMR spectra obtained under proton decoupling spreads over a larger chemical shift range, and this makes it easier to identify the sugar resonances in honey [10]. To our best knowledge, the relevant scientific reports about using ^13^C NMR for the analysis of sugars in honey are still limited. Hence, the major sugar molecules of stingless bee honey, including mono- and di-saccharides, and their respective tautomers were identified and quantified in this study using ^13^C NMR spectroscopy.

Reducing sugars undergo mutarotation and form two or more species known as tautomers. Mutarotation happens when the hemiketal ring opens and closes and with α or β configuration [12,13,14]. For instance, d-glucose in aqueous solution appears in two forms, which are α-d-glucopyranose and β-d-glucopyranose; fructose appears in four forms in aqueous solution, namely α-d-fructopyranose, β-d-fructopyranose, α-d-fructofuranose, and β-d-fructofuranose. However, the composition of tautomers varies for each respective reducing sugar in different solvents [12,13,14]. Hence, accurate determination of the composition of tautomers of raw honey in aqueous solution is crucial. Table 1 shows the composition of the tautomers in stingless bee honey, determined by ^13^C NMR spectroscopy, which is in agreement with the literature [10,11,12,13]. Both glucose and fructose are the major monosaccharides found in honey formed by the hydrolysis of sucrose by enzyme invertase [15]. The reducing sugar content in stingless bee honey samples ranging from 73% to 73.61% also met the requirement set by the Department of Standards Malaysia, which is not more than 85.0% [9]. Honeydew honey was found to possess very minimal sucrose to be integrated by ^13^C NMR spectroscopy and sucrose content obtained from blossom honey in this study (0.40%) met the established requirement, which is not more than 9.5%. The values obtained by other authors in stingless bee honeys also met the requirements [16,17]. However, the presence of trehalulose in tested stingless bee honey samples was not detected by ^13^C NMR spectroscopy. The chemical shift for C1 of α-d-glucopyranose in trehalulose (99.3–101.3 ppm) [18,19,20] was not observed in stingless bee honey samples in this study. Moreover, the obtained chemical shift for C1 of α-d-glucopyranose tautomer was consistent with the value from a standard glucose compound as reported in another study (92.7 ppm) [10]. Hence, contrary to an earlier study [18], such disaccharide could be absent or present only in very low quantity in the tested stingless bee honey samples produced by *H. itama* and *G. thoracica*.

It has been reported that honeydew honey presented a lower value of the sum of glucose plus fructose than blossom honey because blossom honey is usually tested with higher glucose content [21,22]. However, this study and other authors showed different results in which there were no significant differences for both types of honey [23]. Glucose tends to precipitate and crystalize because it is less water soluble than fructose. The tendency of crystallization in honey can be evaluated based on the fructose-to-glucose ratio (F/G) and the glucose-to-moisture ratio (G/M). According to a previous study, F/G not more than 1.14 is associated with honey crystallization, whereas values 1.33 and above indicate a slower crystallization [24]. Other authors suggested that the G/M ratio could be a better indicator for honey crystallization with values not more than 1.7 indicate slow or no crystallizations, while a higher value up to 2 suggests rapid crystallization [21,22,25]. In this study, the F/G values of honeydew and blossom honeys were 1.06 and 1.08, respectively, whereas the G/M values of honeydew and blossom honeys were 1.31 and 1.38, respectively. Hence, these values suggest a slower crystallization of both honeydew and blossom honeys. Although the average value of total sugars in honeydew honey (73%) was lower than blossom honey (74.01%), there was no significant different in the total content of glucose and fructose between honeydew honey and blossom honey. Hence, the major sugar composition is suggested to be consistent among stingless bee honey.

A previous study also stated that honeydew honey is generally darker than the blossom origin honey [22]. The color intensity of raw honey is mainly related to the botanical origin and composition including mineral, pollen, and phenolic compounds [26]. In this study, honeydew honey with darker color (Table 2) was tested with higher mineral content (Table 5) and phenolic compounds (Table 6). The moisture content in stingless bee honey samples ranged from 22.50% to 28.00%, clearly exceeding the limits established by Codex Alimentarius Commission and Council Directive of the European Union, which stipulated the moisture content must be below 20% [6,7]. However, the values still comply with the Malaysian Standard for stingless bee honey established by the Department of Standards Malaysia that the moisture content of stingless bee honey should not exceed 35.0% [9]. The moisture content in stingless bee honey is one of the unique parameters demonstrating the importance of an international legislation geared toward stingless bee honeys. The significant difference in the moisture contents of honey bee honey and stingless bee honey is mainly due to the different strategies used to produce honey. Honey bees remove moisture by using their wings and store honey in sealed honeycombs, while stingless bees do not vaporize honey using their wings, and the honey has contact with the air until the honey pots are fully enclosed [17,27]. Higher moisture content of stingless bee honey than honey bee honey was also shown in other studies [16,28,29]. In addition to that, the difference in moisture content between honeydew honey and blossom honey could be due to a different nectar source [30,31].

Despite several articles reporting no significant difference for moisture content and water activity between honeydew and blossom honeys [21,22], stingless bee honeydew honey in this study was tested to have significantly higher moisture content. Moisture content is highly associated with water activity; water activity is a better indicator of microbial growth because it elucidates the amount of free water available for microorganisms [17]. The values of water activity obtained in this study were lower than previously reported values of 0.76–0.87 from stingless bee honey [17]. Water activity lower than 0.60 is able to prevent microbial growth including osmophilic yeasts; hence, stingless bee honey is generally more susceptible to microbial fermentation [17]. This parameter can be considered to be included in legislation to ensure the stability of stingless bee honey toward microbial fermentation. However, there was no significant difference in water activity between honeydew honey and blossom honey [21]. The total soluble solid in stingless bee honey (72.00–77.50 °Brix) was found to be in agreement with the values reported by previous studies from 60.85 to 72.25 °Brix [17] and from 55.20 to 76.10 °Brix [32]. Although different °Brix values in honey samples could be due to different amounts of sugars, the °Brix value is directly related to the amount of sugar in the honey [24,25]. The content of glucose and fructose is not significantly different between honeydew honey and blossom honey in this study as described above. Hence, it was suggested that lower total soluble solid could be linked to a significantly lower amount of proline and hydroxymethylfurfural found in honeydew honey.

Proline is the major component with 50–85% of the total amino acids in honey [33]. Proline content in honey has to be at least 180 mg/kg to be accepted internationally [16]. All stingless bee honey samples in this study met this requirement with the range of 454.80–670.27 mg/kg. The proline content of stingless bee honey in this study was found to be higher than the reported values by another study, ranging from 276.11 to 498.52 mg/kg [16]. Since the presence of proline in honey is related to its botanical source, it can be suggested that such a variation is due to the botanical preference of bees during nectar collection [34]. However, the high variability of the proline content in honey is unable to differentiate between the honeydew and blossom origins [22,32].

Both electrical conductivity and ash content are reliable parameters to differentiate between honeydew and blossom honeys [22]. Although there is no reference range recommended by Codex Alimentarius Commission and Council Directive of the European Union for the electrical conductivity of stingless bee honey [6,7], the electrical conductivity of stingless bee honey as shown in Table 3 (0.28–0.52 mS/cm) was found to be similar to the previously reported values, ranging from 0.32 to 1.10 mS/cm [28], ranging from 0.35 to 0.76 mS/cm [35], and ranging from 0.30 to 0.67 mS/cm [30]. The ash content of stingless bee honey samples fulfilled the requirement set by the Department of Standards Malaysia, which is not more than 1.00% [9]. Variations in electrical conductivity and ash content among different honey types were suggested due to different botanical sources foraged by the bees [28]. In this study, the values of both electrical conductivity and ash content showed great variability among botanical origins, whereby values found in honeydew honey were significantly higher than blossom honey, which indicate that honeydew honey is richer in both organic and inorganic substances including minerals (Table 5) and organic acids (Table 4).

Diastase is the major enzyme in honey, followed by invertase and glucose oxidase. Enzyme diastase catalyzes the breakdown of starch into maltose which originated from nectar, exudate, and the bees [36]. It is commonly used as an indicator of honey purity and freshness [37]. This enzyme is sensitive to heat and prolonged poor storage conditions; hence, it can be also used to monitor the deterioration of honey [8]. Diastase activity is commonly used in Europe as the indication of freshness of honey. To date, there is no standard for the diastase level of stingless bee honey. The diastase level in stingless bee honey samples reported in this study was rather low, ranging from 1.83 to 3.04 Schade units as compared with the diastase levels reported by a previous study ranging from 0.9 to 23.0 Schade units [38]. Low diastase level in honey samples could be due to acidic pH and hot climate. Diastase activity could be inactivated at pH values lower than 5.3 [39]. Therefore, low diastase level could be explained due to high acidity, as the pH values of stingless bee honey samples in this study range from 3.16 to 3.54. The higher temperature in tropical regions may also diminish the diastase number in honey [8]. Although a previous study showed there were no significant differences in the mean values of diastase activity between honeydew and blossom honeys [21], the honeydew honey was found to have significant higher enzymatic activity than blossom honey in this study.

Hydrogen peroxide is known to be a strong predictor for the antimicrobial activity in honey. To date, there is no legislation available for this parameter. The hydrogen peroxide level of stingless bee honey samples in this study ranged from 111.07 to 192.82 µmol/L which was slightly higher than the hydrogen peroxide level of stingless bee honey reported by another study ranging from 91.50 to 155.80 µmol/L [40]. A significantly higher level of hydrogen peroxide in honeydew honey may be associated with greater antimicrobial activity [41]. Although hydrogen peroxide is an important inhibitory factor of microbial growth, it is not the sole parameter that determines the antimicrobial potency of honey [41]. Although honey with no hydrogen peroxide had little or no antimicrobial activity, some honey samples also failed to exhibit antimicrobial activity despite having a relatively high level of hydrogen peroxide [42]. Studies showed that phenolic compounds and their interaction with hydrogen peroxide are the key factors responsible for the antimicrobial activity of honey [42,43,44]. The information about hydrogen peroxide level in stingless bee honey is still very limited. Hence, more studies are required to investigate the correlation of hydrogen peroxide with the antimicrobial effects of stingless bee honey.

As shown in Table 4, the pH values (from 3.16 to 3.54) of stingless bee honey were in compliance with the Malaysian Standard, in which they should fall between 2.5 and 3.8 [9]. The pH values of stingless bee honey also were similar to the values reported by other studies, in the range of 3.29–3.71 and 3.22–4.03, respectively [45,46]. The high free acidity found in stingless bee honey (60.67–95.33 meq/kg) appears to be a specific characteristic, but stingless bee honey is not included in the international standards for honey. This is due to the presence of organic acids, particularly gluconic acid and acetic acid [22]. Another article stated honeydew honey produced by honey bee showed a significantly higher pH value and acidity than blossom honey [21]. In this study, the free acidity of honeydew honey produced by stingless bee was also significantly higher than the blossom honey, but the pH value was not significantly higher.

Organic acids are minor constituents in honey, accounting for less than 0.5% of the fresh weight of honey. Still, the acidity of honey is strongly related with the organic acids in honey, which are derived from glucose oxidase enzymatic pathway, such as gluconic acid or from microbial fermentation, such as acetic acid [30,47]. Similarly, another study also stated that gluconic acid was the major organic acid in stingless bee honey with the range from 0.07 to 1.48 g/kg [17]. Such findings indicate the importance of gluconic acid in the acidity characteristic of honey. Studies showed that honey with a higher water content can promote the enzymatic activity of glucose oxidase to produce gluconic acid [48,49]. A wide variation of gluconic acid content is possible due to different amount of glucose and glucose oxidase enzymatic activity in honey [17]. The values of acetic acid evaluated in this study were also within the range of values reported by a previous study, ranging from 0.01 to 0.39 g/kg [17]. The higher acidity of honeydew honey in this study could be explained with significant higher level of acetic acid than blossom honey. Excessive acetic acid content is a good indicator of deterioration due to microbial fermentation, although acetic acid is generally found in most honeys [50]. To date, there is no study of normal and fermentation levels of acetic acid in honey. Other than gluconic and acetic acids, the acidity of honey is also contributed to by other organic acids such as succinic, formic, and malic acids [17,51]. Despite the contributions of organic acids to organoleptic properties, especially flavor and to physicochemical properties such as pH and free acidity, information about organic acids of honey is still very limited.

Hydroxymethylfurfural (HMF) content is one of the parameters used to assess the deterioration of honey, because HMF is produced from the degradation of sugars present in honey [8]. HMF is formed slowly during storage or at a faster rate if the honey is heated [15]. The HMF content of stingless bee honey samples were in accordance with the reference value established by the Department of Standards Malaysia for stingless bee honey at maximum level of 30 mg/kg [9]. Such expected values indicating the honey samples were harvested and stored properly, without undergoing any heating process or exposure to high ambient temperature during storage [32].

Table 5 shows that Na is the most abundant element in all stingless bee honey samples, ranging from 223.87 to 326.75 mg/kg, followed by K ranging from 165.27 to 302.40 mg/kg. To our knowledge, this is the first report that Na but not K is the most abundant element detected in honey. Other studies stated K was the most abundant amount of mineral element detected ranging from less than 105.50 to 761.22 mg/kg [32,52]. Na content recorded in the study was found to be within the range values of other studies as well, ranging from 73.00 to 589.46 mg/kg in stingless bee honey [32,52]. Other major mineral elements in stingless bee honey are Ca and Mg, ranging from 37.72 to 70.73 mg/kg and from 32.77 to 59.95 mg/kg, respectively. Other studies also reported similar values for Ca and Mg, ranging from 11.20 to 352.00 mg/kg and from 4.10 to 173.00 mg/kg, respectively [32,52]. Other mineral elements including Fe, Zn, Mn, and Cr were present in low quantities in stingless bee honey [32,52]. Instead of bee types, botanical and geographical origins are the primary factors of mineral content [53]. A previous study also found that the total mineral elements of honeydew honey including K, Mg, and Ca were significantly higher than those in blossom honey [54]. Significantly higher total mineral elements in honeydew honey were also reflected in electrical conductivity and ash content analyses (Table 3) in this study.

Analyzing the data, it can be noted that the data of certain parameters including moisture content, free acidity and diastase activity are considerably different between stingless bee honey and international legislations for honey bee honey specifically *Apis mellifera* (Codex Alimentarius and Council Directive of the European Union). Such data were not compliant with the established standard limits. Therefore, the current legislations should review the standard limits to fit the majority of studied stingless bee honeys from tropical and subtropical regions.

The total phenolic content of stingless bee honey (56.78–120.06 mg GAE/kg) as shown in Table 6 was generally higher than the previous reported values, from 27.33 to 55.86 mg GAE/kg; hence, higher ferric reducing power was observed in this study ranging from 1.33 to 3.58 mmol Fe(II)/kg compared with 0.54–1.64 mmol Fe(II)/kg [24]. Interestingly, another study reported the antioxidant properties of honey bee honey were found to be higher (total phenolic content: 409.73–475.68 mg GAE/kg; ferric reducing power: 5.00–5.26 mmol Fe(II)/kg) than a stingless bee honey that was produced by *Melipona* sp. (total phenolic content: 354.03–386.09 mg GAE/kg; ferric reducing power: 4.17–4.39 mmol Fe(II)/kg), but lower than another stingless bee honey which was produced by *Hypotrigona* sp. (total phenolic content: 375.82–522.91 mg GAE/kg; ferric reducing power: 6.65–6.69 mmol Fe(II)/kg) [22]. Hence, it can be said that there is no definite comparison outcome regarding the antioxidant properties in honey produced by different bee types. Since phenolic compounds in honey originate from plants, the phenolic content is greatly affected by the nectar source harvested by the bees [55]. In this study, honeydew honey was found not only to have significantly higher total phenolic content, and also significantly higher antioxidant capacities including ABTS, DPPH and superoxide radical scavenging activities, peroxyl radical inhibition, iron chelation, and ferric reducing power than blossom honey. Several authors also stated honeydew honey, which is usually darker in color possesses higher antioxidant activities [22,51]. A previous study also found that the mean values of total phenolic content (105.42 mg GAE/100 g), DPPH radical scavenging activity (41.54 mg AAE/100 g), and ferric reducing power (861.06 µmol Fe(II)/100 g) of honeydew honey produced by honey bees were higher than blossom honey, 60.50 mg GAE/100 g, 18.60 mg AAE/100 g, and 354 µmol Fe(II)/100 g, respectively [8]. There are different methods available to assess the antioxidant activity of honey; hence, it is necessary to use several tests to obtain good reliability [22].

PCA was performed to analyze the physicochemical and antioxidant properties of stingless bee honey samples. As shown in Table 7, this analysis managed to highlight the most suitable parameters to be used in the differentiation of honey samples based on botanical origin. Moreover, this statistical analysis was able to differentiate stingless bee honey samples into two distinctive clusters, which are honeydew and blossom origins (Figure 1). Hence, based on the correlation coefficient, it was suggested that parameters including ash content, hydrogen peroxide, free acidity, total mineral elements, K, Mg, Ca, total phenolic compounds, and ferric reducing power are able to differentiate stingless bee honey between honeydew and blossom origins. PCA was also employed in a previous study to differentiate the honeybee honey into honeydew and blossom types [8]. In their study, free acidity, total phenolic compounds, and ferric reducing power plus electrical conductivity, glucose level, and DPPH scavenging activity were suggested to be the suitable parameters.

## 4. Materials and Methods

### 4.1. Honey Samples

A total of 23 stingless bee honey samples (Table 8) produced by *Heterotrigona itama* (Figure S5a) and *Geniotrigona thoracica* (Figure S5b) were harvested from different areas in the jungle and secondary forest of Southern Negeri Sembilan, Northern Johor, and South Western Pahang in peninsular Malaysia (Figure S6). Raw honey samples were manually filtered and bottled without processing and heat treatment.

### 4.2. Physicochemical Properties

For ^13^C NMR analysis, 0.2 moles of each model compound (glucose, fructose, and sucrose) were prepared in deuterated water (D_2_O). Isoglucose (45% glucose and 55% fructose) and artificial honey (41.27% glucose, 50.79% fructose, and 7.94% sucrose) were prepared with D_2_O. Each honey sample was prepared by dissolving 200 μL (~260 mg) of honey in 300 μL of D_2_O. For internal reference, 0.2 moles of 1,4-dioxane (δ 67.19) was used for quantification. All samples were left overnight to fully equilibrate prior to analysis. ^13^C NMR analysis was conducted on JEOL JNM-ECX400 spectrometer operating at 100 MHz for carbon-13 nuclei. ^13^C NMR spectra of model compounds were obtained with 90° pulse width 7.25 μs: relaxation delay 2 s, 1000–2000 scans, and four pre-scans. For the isoglucose, artificial honey, and honey samples, the number of scans were increased to 10,000, to achieve a better resolution and sensitivity.

The assignment of ^13^C NMR chemical shifts of all sugar model compounds including glucose, fructose, and sucrose and their respective tautomers was completed in the chemical shifts reported in the literature [10,11]. The integration of ^13^C signals allows the direct quantitative determination of tautomers of glucose, fructose, and sucrose. The concentration of each sugar tautomer was calculated based on the signals that were unique for this particular tautomer, and overlapped signals were not used for quantification [10,11]. The quantification of the sugar molecules was achieved upon integration of nonoverlapping signals with the known concentration of 1,4-dioxane, for the internal standard. The applicability of ^13^C NMR method to quantify sugar molecules was validated by correlating the amount of glucose and fructose in isoglucose, and the amount of glucose, fructose, and sucrose in the artificial honey determined by ^13^C NMR with the actual weighed amount. The masses of all sugar compounds were calculated in g/100 g, taking into account the moisture content in stingless bee honey.

#### 4.2.1. Color Intensity

The absorbance of filtered honey solution (50%, *w*/*v*) was measured at 450 and 720 nm, and the difference in the absorbance readings is expressed as mAU [56].

#### 4.2.2. Total Soluble Solid and Moisture Content

The refractive index of honey was measured against distilled water using a refractometer (Atago, Japan). The reading was recorded in percent °Brix. Moisture content (%, g/100 g) of honey was calculated [57].

#### 4.2.3. Water Activity

The water activity (aw) of honey was measured using a water activity meter (Novasina, Switzerland).

#### 4.2.4. Proline

Honey solution (0.05 g/mL) was mixed with 50% (*v*/*v*) formic acid and 3% (*w*/*v*) ninhydrin solution, incubated in a boiling water bath for 15 min then at 70 °C for another 10 min, and added with 50% (*v*/*v*) 2-propanol. The mixture was left at room temperature before the absorbance was measured at 510 nm. A standard curve was constructed using proline solution (100–500 µg/mL), and the proline level was calculated based on the equation obtained from the standard curve. The final value of proline content (mg/kg) was determined [58].

#### 4.2.5. Electrical Conductivity

According to harmonized methods of the International Honey Commission [56], electrical conductivity of honey solution (0.2 g/mL) was measured using a multiparameter tester (Oakton Instruments, Vernon Hills, IL, USA), and the result was expressed as millisiemens per centimeter (mS/cm).

#### 4.2.6. Ash Content

Two grams of honey was put in a porcelain crucible and dried in an oven at 110 °C for 4 h. The honey was ashed in an electrical furnace (Nabertherm, Germany) at 600 °C for 6 h and weighed. The ash content % (g/100 g) was calculated [58].

#### 4.2.7. Diastase

Honey solution (0.05 g/mL in 100 mM sodium maleate buffer) was incubated at 40 °C water bath for 5 min. An Amylazyme tablet (Megazyme, Ireland) was added into the sample, incubated for another 10 min and added with Trizma base solution, and left at room temperature for 5 min. The mixture was filtered, and the absorbance of sample solution was measured at 590 nm. The diastase number (DN) of honey was calculated.

#### 4.2.8. Hydrogen Peroxide

Each honey sample and hydrogen peroxide standard (7.81–1000 µmol/L) was mixed individually with a working reagent that was composed of ammonium ferrous (II) sulfate, sulfuric acid, sorbitol, and xylenol orange. The mixture was incubated at room temperature for 20 min, and the absorbance was determined at 595 nm. The concentration of hydrogen peroxide (µmol/L) in each honey was calculated with reference to the equation obtained from the standard curve (Thermo Fisher Scientific Inc., Waltham, MA, USA).

#### 4.2.9. pH

A solution containing 10 g of honey dissolved in 75 mL of distilled water was analyzed using a calibrated pH meter (Sartorius, Germany).

#### 4.2.10. Free Acidity

Ten grams of honey was dissolved in 75 mL of distilled water. Titration to pH 8.30 was completed using a standardized 0.1 M NaOH solution. Free acidity (meq/kg) was calculated [58].

#### 4.2.11. d-gluconic Acid

Each honey and standard solution was individually diluted with distilled water, added with buffer (pH 7.6) plus sodium azide (0.02% *w*/*v*) and NADP^+^/ATP solution. Next, 6-phosphogluconate dehydrogenase suspension was added. After 4 min, the absorbance was read at 340 nm. Lastly, gluconate kinase suspension was added. The absorbance was read again at 340 nm after 6 min. The D-gluconic acid level (g/L) was calculated (Megazyme, Ireland). The final value was expressed as g/kg of honey.

#### 4.2.12. Acetic Acid

Each honey and standard solution was individually diluted with distilled water, added with buffer (pH 7.6), sodium azide (0.02% *w*/*v*) and NADP/ATP/phosphoenolpyruvate (PEP)/polyvinylpyrrolidone (PVP) solution. Next, coenzyme A solution and a mixture composed of d-lactate dehydrogenase, phosphotransacetylase, and pyruvate kinase were added. After 2 min, the absorbance was read at 340 nm. Lastly, acetate kinase suspension was added. The absorbance was read again at 340 nm after 4 min. The acetic acid level (g/L) was calculated (Megazyme, Ireland). The final value was expressed as g/kg of honey.

#### 4.2.13. Hydroxymethylfurfural (HMF)

Carrez solution I (150 mg/mL potassium ferrocyanide) and Carrez solution II (300 mg/mL zinc acetate) were added into 0.2 g/mL of honey solution. The absorbance was measured at 284 and 336 nm. HMF content of honey (mg/kg) was calculated [59].

#### 4.2.14. Mineral Content

Together with standards, the quantitative determination of minerals, including Na, K, Mg, Ca, Fe, Zn, Mn, and Cr, in each honey was analyzed with flame atomic absorption spectroscopy (AAS) (Agilent Technologies, Santa Clara, CA, USA). Honey was digested with 70% nitric acid and 30% hydrogen peroxide overnight and then heated at 100 °C for 10 min before analysis. The concentration of each mineral element (mg/kg) was obtained [60].

### 4.3. Antioxidant Properties

#### 4.3.1. Total Phenolic Compounds

Each honey solution (0.2 g/mL) and gallic acid standard solution (200–1000 µg/mL) was mixed with Folin and Ciocalteu’s phenol reagent. After 3 min, 10% (*w*/*v*) sodium carbonate solution was added. The reaction mixture was incubated in the dark at room temperature for 90 min. The absorbance of each reaction mixture was read at 725 nm. The final value was calculated and expressed in milligram gallic acid equivalents per kg of sample (mg GAE/kg) [61].

#### 4.3.2. ABTS (2,2′-azino-bis(3-ethylbenzothiazoline-6-sulfonic acid)) Radical Scavenging Activity

The ABTS radical cation (ABTS^+^) solution (absorbance of 0.70 ± 0.02 at 734 nm) was incubated in the dark at room temperature for 12–16 h before use. The ABTS^+^ solution was added into honey solution (0.2 g/mL), and the reduction of absorbance was determined after 6 min. The free radical scavenging activity (% RSA) was attained [35].

#### 4.3.3. DPPH (2,2-diphenyl-1-picrylhydrazyl) Radical Scavenging Activity

Honey solution (0.2 g/mL) was added with methanolic solution containing DPPH radicals (0.024 mg/mL), and the absorbance of the mixture was read at 517 nm after 15 min in the dark at room temperature. The free radical scavenging activity (% RSA) was attained [62].

#### 4.3.4. Superoxide Anion Radical Scavenging Activity

The reaction started with peroxymonosulfate solution (60 μM) added with superoxide radical solution (nitroblue tetrazolium, 150 μM and NADH, 468 μM) and honey solution (0.2 g/mL). After incubation at room temperature for 5 min, absorbance was read at 560 nm. The free radical scavenging activity (% RSA) was attained [63].

#### 4.3.5. Peroxyl Radical Inhibitory Activity

Honey solution (0.2 g/mL) and Trolox standard solution were added with fluorescein solution, followed with incubation for 30 min at 37 °C. Then, peroxyl radical solution was added. The absorbance of each honey and standard was read with an excitation wavelength of 480 nm and an emission wavelength of 520 nm, every 5 min for a total of 60 min. By using the calculated area under the curve (AUC), the antioxidant activity of honey was calculated and expressed in micromoles Trolox equivalent per liter sample (µmol TE/L) (Cell Biolabs, San Diego, CA, USA).

#### 4.3.6. Iron Chelating Activity

A reaction mixture was prepared after the addition of honey solution (0.2 g/mL) into 0.10 mM ferrous sulfate and 0.25 mM ferrozine. After standing for 10 min at room temperature, the absorbance was read at 562 nm. The chelating activity (%) was attained [63].

#### 4.3.7. Ferric Reducing Activity

FRAP reagent was added into honey solution (0.2 g/mL) and ferrous sulfate standard solution (0.2–1.0 mmol/L), individually. After the reaction mixture was incubated at 37 °C for 4 min, the absorbance was read at 593 nm. The FRAP value (mmol Fe [II]/kg) was attained [64].

### 4.4. Chemometric Analysis

Each honey was analyzed in triplicates and conducted at 25 °C unless stated otherwise. The data were expressed as means ± standard deviation. Independent t-test was performed to determine the mean value differences at level of significance of 0.05 between honeydew and blossom origins. Principal component analysis (PCA) was employed to interpret interdependence and visualize relatedness between data. The software Microsoft Excel Analyse-it Standard Edition v5.50 was used to perform the statistical analyses.

## 5. Conclusions

This study demonstrated the potential of ^13^C NMR spectroscopy to identify and quantify the major sugar molecules in stingless bee honey samples. Consistent major sugar composition observed in honeydew and blossom honey samples could be useful for the identification of stingless bee honey. Furthermore, several parameters including ash content, hydrogen peroxide, free acidity, total mineral elements, K, Mg, Ca, total phenolic compounds, and ferric reducing power were identified with principal component analysis that can differentiate stingless bee honey samples based on botanical origin. The chemometric analysis demonstrated the potentials of using these parameters to evaluate the authenticity of stingless bee honey in Malaysia. This is the first report characterizing stingless bee honey that collected over the recent three years for the differentiation of botanical origins using chemometrics. Obtained data could be useful for legislations for consideration to review the existing standard limits to fit the stingless bee honeys from tropical and subtropical regions.

## Figures and Tables

**Table 1:** Quantification of sugar tautomers present in stingless bee honey samples using ^13^C NMR spectroscopy.

Sugar	Honeydew Honey			Blossom Honey		
	Tautomer %	Average Integration Value	g/100 g	Tautomer %	Average Integration Value	g/100 g
α-d-glucopyranose (α-GP)	41.70	0.2825	14.41 ± 0.42	40.50	0.2475	14.45 ± 0.31
β-d-glucopyranose (β-GP)	58.30	0.3950	20.99 ± 0.62	59.50	0.3688	20.96 ± 0.52
Total glucose			35.39 ± 0.20			35.36 ± 0.76
α-d-fructopyranose (α-FP)	2.59	0.0188	1.02 ± 0.05	4.17	0.0278	1.60 ± 0.01
β-d-fructopyranose (β-FP)	69.59	0.5050	25.99 ± 0.40	68.99	0.4600	26.19 ± 0.14
α-d-fructofuranose (α-FF)	6.01	0.0436	2.29 ± 0.02	6.20	0.0414	2.39 ± 0.01
β-d-fructofuranose (β-FF)	21.81	0.1583	8.32 ± 0.12	20.65	0.1378	8.08 ± 0.04
Total fructose			37.61 ± 0.20			38.25 ± 0.21
Total sucrose	-	-	-	-	0.0038	0.40 ± 0.57
Fructose-to-glucose ratio (F/G)			1.06 ± 0.01			1.08 ± 0.03
Glucose-to-moisture ratio (G/M)			1.31 ± 0.01			1.38 ± 0.03

Each total content of glucose and fructose between honeydew honey and blossom honey was not significantly different at *p* < 0.05.

**Table 2:** The maturity parameters of honey samples.

Honey Sample	Color Intensity (mAU)	Moisture (%, g/100 g)	Water Activity	Total Soluble Solid (°Brix)	Proline (mg/kg)
S1	180 ± 0	27.30 ± 0	0.62 ± 0	72.70 ± 0	537.13 ± 6.86
S2	190 ± 0	27.13 ± 0.06	0.60 ± 0	72.87 ± 0.06	513.13 ± 5.00
S3	200 ± 0	26.40 ± 0	0.62 ± 0	73.60 ± 0	522.93 ± 20.01
S4	190 ± 0	26.37 ± 0.06	0.63 ± 0	73.63 ± 0.06	542.37 ± 37.50
S5	190 ± 0	26.33 ± 0.06	0.63 ± 0	73.67 ± 0.06	547.43 ± 16.01
S6	150 ± 0	27.87 ± 0.06	0.62 ± 0	72.13 ± 0.06	514.50 ± 3.90
S7	140 ± 0	27.40 ± 0	0.65 ± 0	72.60 ± 0	454.80 ± 11.39
S8	150 ± 0	27.43 ± 0.06	0.65 ± 0	72.57 ± 0.06	546.73 ± 10.83
S9	60 ± 0	23.50 ± 0.10	0.54 ± 0	76.50 ± 0.10	577.00 ± 2.00
S10	80 ± 0	22.57 ± 0.06	0.54 ± 0	77.43 ± 0.06	546.00 ± 2.69
S11	60 ± 0	22.50 ± 0	0.55 ± 0	77.50 ± 0	578.37 ± 4.47
S12	70 ± 0	26.60 ± 0	0.54 ± 0	73.40 ± 0	588.80 ± 35.76
S13	60 ± 0	26.73 ± 0.06	0.57 ± 0	73.27 ± 0.06	514.23 ± 14.70
S14	50 ± 0	24.57 ± 0.06	0.64 ± 0	75.43 ± 0.06	525.27 ± 7.28
S15	50 ± 0	24.30 ± 0	0.63 ± 0	75.70 ± 0	525.70 ± 4.62
S16	70 ± 0	24.20 ± 0	0.63 ± 0	75.80 ± 0	525.10 ± 7.82
S17	250 ± 0	26.20 ± 0	0.64 ± 0	73.80 ± 0	670.27 ± 6.78
S18	250 ± 0	27.20 ± 0	0.67 ± 0	72.80 ± 0	598.50 ± 3.50
S19	240 ± 0	27.10 ± 0	0.67 ± 0	72.87 ± 0.06	612.40 ± 4.25
S20	280 ± 0	27.30 ± 0	0.67 ± 0	72.70 ± 0	627.57 ± 7.10
S21	220 ± 0	27.37 ± 0.06	0.67 ± 0	72.63 ± 0.06	510.67 ± 5.86
S22	250 ± 0	28.00 ± 0	0.69 ± 0	72.00 ± 0	510.47 ± 1.50
S23	210 ± 0	26.97 ± 0.06	0.67 ± 0	73.03 ± 0.06	535.70 ± 4.96
Average	156.09 ± 76.59	26.14 ± 1.64	0.62 ± 0.05	73.85 ± 1.64	548.94 ± 47.74
Honeydew	173.75 ± 22.23	27.03 ± 0.56 *	0.63 ± 0.02	72.97 ± 0.56 *	522.38 ± 32.61 *
Blossom	146.67 ± 92.44	25.67 ± 1.83	0.62 ± 0.06	74.32 ± 1.83	563.11 ± 48.74

*—Significant difference between honeydew honey and blossom honey at *p* < 0.05.

**Table 3:** The purity parameters of honey samples.

Honey Sample	Electrical Conductivity (mS/cm)	Ash Content (%, g/100 g)	Diastase (Schade Unit/g)	Hydrogen Peroxide (µmol/L)
S1	0.36 ± 0	0.18 ± 0.01	3.04 ± 0.08	177.58 ± 19.80
S2	0.39 ± 0	0.16 ± 0.01	2.98 ± 0.05	185.43 ± 7.29
S3	0.42 ± 0	0.15 ± 0	2.45 ± 0.03	187.05 ± 20.39
S4	0.45 ± 0	0.13 ± 0.01	2.51 ± 0.09	177.19 ± 18.63
S5	0.41 ± 0	0.13 ± 0.01	2.71 ± 0.04	189.77 ± 18.09
S6	0.52 ± 0	0.13 ± 0	2.22 ± 0.08	184.07 ± 13.14
S7	0.51 ± 0	0.13 ± 0.01	2.05 ± 0.02	192.82 ± 20.58
S8	0.52 ± 0	0.14 ± 0.01	2.34 ± 0.07	174.18 ± 6.22
S9	0.38 ± 0	0.04 ± 0	2.63 ± 0.59	111.40 ± 1.77
S10	0.35 ± 0	0.04 ± 0	2.09 ± 0.02	111.97 ± 1.82
S11	0.29 ± 0	0.04 ± 0.01	2.25 ± 0.03	118.20 ± 10.78
S12	0.29 ± 0	0.04 ± 0.01	2.15 ± 0.04	112.40 ± 2.50
S13	0.41 ± 0	0.04 ± 0.01	2.30 ± 0.04	112.95 ± 3.49
S14	0.42 ± 0	0.04 ± 0.01	2.17 ± 0.18	119.53 ± 8.88
S15	0.45 ± 0	0.04 ± 0.01	2.39 ± 0.02	114.15 ± 9.31
S16	0.45 ± 0	0.04 ± 0	2.38 ± 0.03	111.07 ± 11.28
S17	0.38 ± 0	0.05 ± 0.01	1.83 ± 0.15	168.37 ± 21.51
S18	0.35 ± 0	0.05 ± 0.01	2.23 ± 0.35	146.17 ± 30.41
S19	0.30 ± 0	0.05 ± 0.01	2.18 ± 0.14	166.73 ± 12.74
S20	0.28 ± 0	0.05 ± 0.01	2.16 ± 0.14	160.17 ± 17.36
S21	0.36 ± 0	0.05 ± 0.01	2.43 ± 0.02	169.63 ± 4.57
S22	0.38 ± 0	0.05 ± 0.01	2.36 ± 0.03	178.61 ± 2.01
S23	0.38 ± 0	0.05 ± 0	2.29 ± 0.08	162.77 ± 21.68
Average	0.39 ± 0.07	0.08 ± 0.05	2.35 ± 0.31	153.57 ± 33.12
Honeydew	0.45 ± 0.06 *	0.14 ± 0.02*	2.54 ± 0.34 *	183.51 ± 15.11 *
Blossom	0.36 ± 0.05	0.04 ± 0.01	2.26 ± 0.24	137.61 ± 28.74

*—Significant difference between honeydew honey and blossom honey at *p* < 0.05.

**Table 4:** The deterioration parameters of honey samples.

Honey Sample	pH	Free Acidity (meq/kg)	Gluconic Acid (g/kg)	Acetic Acid (g/kg)	Hydroxymethylfurfural (mg/kg)
S1	3.26 ± 0.02	89.33 ± 0.58	0.34 ± 0.01	0.09 ± 0.01	19.69 ± 0.04
S2	3.26 ± 0.01	87.33 ± 0.58	0.31 ± 0.01	0.11 ± 0.02	19.41 ± 0.56
S3	3.18 ± 0.02	85.00 ± 1.00	0.38 ± 0.06	0.13 ± 0.03	19.48 ± 0.36
S4	3.16 ± 0.02	93.00 ± 2.00	0.34 ± 0.01	0.12 ± 0.04	17.31 ± 0.81
S5	3.21 ± 0.02	90.33 ± 0.58	0.37 ± 0.03	0.09 ± 0.03	16.62 ± 0.22
S6	3.54 ± 0.01	94.67 ± 0.58	0.71 ± 0.06	0.09 ± 0.03	11.45 ± 0.37
S7	3.54 ± 0.01	92.67 ± 0.58	0.62 ± 0.03	0.08 ± 0.02	14.85 ± 0.32
S8	3.51 ± 0.01	95.33 ± 0.58	0.62 ± 0.04	0.09 ± 0.03	15.64 ± 0.11
S9	3.46 ± 0.01	72.00 ± 1.00	0.57 ± 0.01	0.09 ± 0.03	19.87 ± 0.46
S10	3.51 ± 0.01	70.67 ± 0.58	0.54 ± 0.05	0.16 ± 0.05	19.17 ± 0.38
S11	3.28 ± 0.01	72.00 ± 0	0.58 ± 0.03	0.12 ± 0.02	17.22 ± 0.72
S12	3.27 ± 0.01	78.00 ± 0	0.45 ± 0	0.09 ± 0.02	27.10 ± 0.92
S13	3.22 ± 0.01	75.00 ± 0	0.35 ± 0.01	0.08 ± 0.01	13.13 ± 1.51
S14	3.25 ± 0.02	62.00 ± 2.00	0.56 ± 0.05	0.10 ± 0.02	13.54 ± 0.44
S15	3.17 ± 0.02	60.67 ± 0.58	0.67 ± 0.02	0.10 ± 0.02	14.22 ± 0.41
S16	3.22 ± 0.03	61.67 ± 0.58	0.72 ± 0.06	0.13 ± 0.02	12.71 ± 0.55
S17	3.21 ± 0	80.67 ± 0.58	0.34 ± 0.01	0.02 ± 0.01	20.24 ± 0.46
S18	3.25 ± 0.01	81.67 ± 0.58	0.35 ± 0.03	0.05 ± 0.02	20.39 ± 0.46
S19	3.21 ± 0.01	81.67 ± 0.58	0.43 ± 0.04	0.03 ± 0.01	19.53 ± 0.27
S20	3.28 ± 0.01	85.00 ± 0	0.25 ± 0.01	0.03 ± 0.02	21.10 ± 1.28
S21	3.51 ± 0	86.67 ± 0.58	0.34 ± 0.02	0.04 ± 0.01	24.54 ± 0.04
S22	3.51 ± 0	91.00 ± 1.00	0.30 ± 0.03	0.03 ± 0.02	19.69 ± 0.19
S23	3.50 ± 0	91.00 ± 0	0.65 ± 0.05	0.04 ± 0.01	18.88 ± 1.04
Average	3.33 ± 0.14	81.62 ± 10.70	0.47 ± 0.15	0.08 ± 0.04	18.08 ± 3.74
Honeydew	3.33 ± 0.16	90.96 ± 3.54 *	0.46 ± 0.15	0.10 ± 0.03 *	16.81 ± 2.74 *
Blossom	3.32 ± 0.13	76.64 ± 9.88	0.47 ± 0.15	0.07 ± 0.05	18.76 ± 4.05

*—Significant different between honeydew honey and blossom honey at *p* < 0.05.

**Table 5:** (1) The mineral elements profile of honey samples. (2) The mineral elements profile of honey samples.

**(1)**					
**Honey Sample**	**Na** **(mg/kg)**	**K** **(mg/kg)**	**Mg** **(mg/kg)**	**Ca** **(mg/kg)**	**Fe** **(mg/kg)**
S1	283.40 ± 6.01	298.27 ± 11.06	50.51 ± 0.64	67.27 ± 2.85	12.17 ± 1.13
S2	295.42 ± 1.95	285.92 ± 7.17	55.39 ± 1.29	70.24 ± 2.89	11.82 ± 1.51
S3	326.75 ± 22.74	269.35 ± 40.91	58.19 ± 0.42	69.76 ± 2.96	12.86 ± 0.35
S4	305.63 ± 14.03	296.93 ± 6.39	59.95 ± 0.38	65.54 ± 3.05	14.29 ± 0.89
S5	312.14 ± 25.09	274.47 ± 28.46	54.54 ± 0.49	70.73 ± 0.96	12.77 ± 1.22
S6	300.13 ± 9.81	302.40 ± 4.27	52.64 ± 0.86	66.43 ± 3.83	13.77 ± 1.97
S7	308.57 ± 10.66	285.60 ± 14.20	52.99 ± 0.44	69.03 ± 2.43	12.26 ± 0.86
S8	316.87 ± 32.21	273.73 ± 59.26	54.29 ± 1.46	69.77 ± 2.36	12.50 ± 0.56
S9	256.60 ± 27.52	190.69 ± 11.00	42.02 ± 1.89	47.23 ± 5.72	12.34 ± 0.80
S10	242.68 ± 12.61	178.61 ± 14.91	35.95 ± 3.03	37.72 ± 0.79	10.41 ± 0.74
S11	223.87 ± 16.54	167.20 ± 11.02	32.77 ± 1.04	38.24 ± 2.70	10.73 ± 1.50
S12	263.65 ± 11.12	166.67 ± 9.62	34.57 ± 3.59	47.45 ± 2.12	10.68 ± 1.21
S13	243.53 ± 48.50	165.27 ± 14.55	42.09 ± 5.21	45.45 ± 4.39	10.90 ± 1.57
S14	286.20 ± 6.91	191.37 ± 6.25	42.53 ± 5.12	53.90 ± 7.37	12.73 ± 0.81
S15	277.03 ± 23.49	204.00 ± 16.52	47.41 ± 4.04	58.13 ± 2.75	12.50 ± 0.66
S16	283.70 ± 5.79	179.83 ± 17.82	44.78 ± 2.69	63.27 ± 5.16	11.87 ± 0.61
S17	278.83 ± 12.71	223.65 ± 14.98	44.58 ± 1.51	61.75 ± 2.08	11.26 ± 0.92
S18	278.59 ± 28.74	232.70 ± 16.22	46.36 ± 1.35	63.73 ± 0.50	11.38 ± 0.78
S19	260.30 ± 43.61	209.05 ± 18.20	44.82 ± 2.06	63.39 ± 1.17	10.71 ± 0.93
S20	263.88 ± 28.77	224.23 ± 13.89	40.25 ± 1.85	63.99 ± 2.93	12.09 ± 0.70
S21	275.60 ± 25.84	189.27 ± 12.66	46.13 ± 5.16	70.30 ± 2.09	12.33 ± 1.46
S22	274.60 ± 7.45	184.07 ± 11.60	50.83 ± 4.49	69.33 ± 0.90	10.57 ± 1.10
S23	292.27 ± 6.55	179.93 ± 1.15	47.02 ± 2.34	63.77 ± 6.77	11.57 ± 0.80
Average	280.45 ± 31.09	224.92 ± 51.17	46.98 ± 7.56	60.71 ± 10.70	11.94 ± 1.34
Honeydew	306.11 ± 19.79 *	285.83 ± 26.51 *	54.81 ± 3.00 *	68.60 ± 2.96 *	12.80 ± 1.26 *
Blossom	266.76 ± 27.16	192.44 ± 24.00	42.81 ± 5.68	56.51 ± 10.98	11.47 ± 1.14
**(2)**					
**Honey Sample**	**Zn** **(mg/kg)**	**Mn** **(mg/kg)**	**Cr** **(mg/kg)**	**Cu + Al (mg/kg)**	**Total Mineral Elements** **(mg/kg)**
S1	3.40 ± 0.26	0.44 ± 0.14	0.42 ± 0.08	<LOQ	715.88 ± 7.39
S2	3.74 ± 0.32	0.29 ± 0.21	0.66 ± 0.38	<LOQ	723.47 ± 5.73
S3	3.78 ± 0.38	0.59 ± 0.25	0.27 ± 0.20	<LOQ	741.56 ± 38.62
S4	3.63 ± 1.10	0.75 ± 0.16	0.44 ± 0.10	<LOQ	747.15 ± 16.63
S5	2.66 ± 0.58	0.63 ± 0.04	0.27 ± 0.20	<LOQ	728.21 ± 54.06
S6	3.88 ± 0.30	0.63 ± 0.06	0.63 ± 0.32	<LOQ	740.52 ± 3.59
S7	4.25 ± 0.28	0.72 ± 0.28	0.53 ± 0.40	<LOQ	733.95 ± 2.96
S8	4.14 ± 0.46	0.67 ± 0.15	0.60 ± 0.26	<LOQ	732.56 ± 26.36
S9	3.47 ± 0.65	0.62 ± 0.13	0.61 ± 0.11	<LOQ	553.58 ± 13.19
S10	3.41 ± 0.19	0.54 ± 0.07	0.56 ± 0.12	<LOQ	509.88 ± 9.36
S11	2.27 ± 0.85	0.42 ± 0.14	0.45 ± 0.06	<LOQ	475.94 ± 24.77
S12	2.98 ± 0.11	0.40 ± 0.13	0.51 ± 0.07	<LOQ	526.91 ± 15.85
S13	2.60 ± 0.38	0.52 ± 0.06	0.67 ± 0.11	<LOQ	511.02 ± 44.73
S14	3.03 ± 0.39	0.68 ± 0.10	0.82 ± 0.07	<LOQ	591.27 ± 13.90
S15	2.71 ± 0.33	0.69 ± 0.12	0.42 ± 0.12	<LOQ	602.90 ± 42.12
S16	3.31 ± 0.44	0.67 ± 0.12	0.61 ± 0.36	<LOQ	588.03 ± 20.94
S17	2.39 ± 0.78	0.45 ± 0.19	0.53 ± 0.25	<LOQ	623.44 ± 17.96
S18	2.00 ± 0.92	0.37 ± 0.05	0.63 ± 0.12	<LOQ	635.77 ± 31.56
S19	1.81 ± 0.19	0.39 ± 0.18	0.48 ± 0.09	<LOQ	590.96 ± 54.17
S20	1.76 ± 0.24	0.42 ± 0.27	0.54 ± 0.04	<LOQ	607.17 ± 19.59
S21	2.94 ± 0.24	0.60 ± 0.17	0.73 ± 0.16	<LOQ	597.89 ± 46.93
S22	3.32 ± 0.49	0.65 ± 0.05	0.59 ± 0.08	<LOQ	593.96 ± 9.66
S23	3.09 ± 0.17	0.80 ± 0	0.79 ± 0.17	<LOQ	599.24 ± 8.09
Average	3.07 ± 0.81	0.56 ± 0.19	0.55 ± 0.21	-	629.19 ± 88.03
Honeydew	3.68 ± 0.65 *	0.59 ± 0.21	0.48 ± 0.27	-	732.91 ± 23.96 *
Blossom	2.74 ± 0.70	0.55 ± 0.17	0.60 ± 0.17	-	573.86 ± 51.74

*—Significantly different between honeydew honey and blossom honey at *p* < 0.05. LOQ—Limits of quantitation.

**Table 6:** The antioxidant properties of honey samples.

Honey Sample	Total Phenolic Compounds (mg GAE/kg)	ABTS Radical Scavenging Activity (%)	DPPH Radical Scavenging Activity (%)	Superoxide Radical Scavenging Activity (%)	Peroxyl Radical Inhibition (µmol TE/g)	Iron Chelation (%)	Ferric Reducing Power (mmol Fe(II)/kg)
S1	105.60 ± 1.63	59.13 ± 1.87	34.83 ± 0.56	76.60 ± 0.36	5.47 ± 0.04	17.38 ± 1.53	3.20 ± 0.02
S2	105.60 ± 4.37	63.28 ± 0.41	36.03 ± 0.33	77.51 ± 0.34	5.61 ± 0.30	14.29 ± 0.44	3.18 ± 0.03
S3	120.06 ± 1.29	70.90 ± 0.36	36.26 ± 0.25	76.00 ± 0.10	5.81 ± 0.16	16.02 ± 0.81	3.31 ± 0.02
S4	109.74 ± 1.03	68.17 ± 0.82	34.52 ± 1.27	75.30 ± 0.26	5.61 ± 0.12	16.08 ± 0.47	3.29 ± 0.02
S5	113.59 ± 1.69	68.69 ± 0.66	37.37 ± 1.50	76.50 ± 0.40	6.12 ± 0.33	16.18 ± 0.70	3.58 ± 0.31
S6	95.39 ± 2.63	55.68 ± 0.70	35.44 ± 0.40	73.98 ± 0.03	5.88 ± 0.32	22.51 ± 0.93	3.06 ± 0.04
S7	83.28 ± 16.36	58.00 ± 0.70	35.25 ± 0.69	76.02 ± 0.79	5.48 ± 0.24	21.74 ± 1.87	2.96 ± 0.05
S8	99.51 ± 1.62	61.33 ± 0.58	35.49 ± 0.93	76.92 ± 0.24	5.70 ± 0.33	27.29 ± 1.52	2.87 ± 0.05
S9	65.58 ± 2.03	53.89 ± 0.45	36.14 ± 0.42	70.80 ± 0.80	4.58 ± 0.18	16.43 ± 1.56	1.33 ± 0.01
S10	73.54 ± 2.11	62.07 ± 0.53	32.62 ± 2.17	71.07 ± 0.67	4.90 ± 0.17	13.08 ± 0.91	1.71 ± 0.01
S11	62.25 ± 0.94	53.44 ± 0.68	35.48 ± 2.20	69.73 ± 0.70	5.19 ± 0.27	10.83 ± 0.26	1.64 ± 0.03
S12	75.51 ± 2.25	54.33 ± 0.31	34.33 ± 0.31	74.13 ± 2.80	5.18 ± 0.20	8.26 ± 1.23	1.63 ± 0.04
S13	61.01 ± 2.22	44.77 ± 0.66	34.67 ± 0.51	73.87 ± 2.34	5.97 ± 0.20	10.54 ± 0.56	1.56 ± 0.04
S14	57.35 ± 0.98	51.28 ± 0.24	33.38 ± 0.72	74.06 ± 1.43	5.60 ± 0.17	12.91 ± 1.67	1.57 ± 0.05
S15	56.78 ± 2.24	56.38 ± 0.70	32.84 ± 1.74	75.03 ± 0.45	4.85 ± 0.27	10.18 ± 1.02	1.83 ± 0.01
S16	59.32 ± 0.85	51.82 ± 0.35	33.09 ± 0.51	72.45 ± 1.33	4.44 ± 0.10	11.62 ± 0.69	1.56 ± 0.04
S17	97.04 ± 1.45	58.37 ± 0.47	31.59 ± 0.80	71.13 ± 0.81	5.61 ± 0.24	12.37 ± 0.68	1.62 ± 0.08
S18	99.94 ± 2.69	60.44 ± 0.87	33.44 ± 1.55	75.07 ± 0.50	5.60 ± 0.18	14.79 ± 0.26	2.25 ± 0.05
S19	94.67 ± 2.05	56.01 ± 0.22	31.56 ± 0.69	79.36 ± 0.55	5.65 ± 0.11	19.15 ± 0.48	1.90 ± 0.07
S20	101.13 ± 1.21	62.00 ± 0.26	32.83 ± 1.80	73.73 ± 1.42	5.66 ± 0.06	11.78 ± 0.38	2.20 ± 0.02
S21	78.22 ± 0.59	66.28 ± 0.48	33.16 ± 0.69	77.22 ± 0.70	5.64 ± 0.20	12.89 ± 0.25	2.00 ± 0.01
S22	88.14 ± 0.85	66.74 ± 0.82	32.17 ± 0.69	78.89 ± 1.40	5.51 ± 0.11	15.07 ± 0.61	2.29 ± 0.08
S23	77.11 ± 2.68	56.92 ± 0.58	31.90 ± 1.63	78.90 ± 1.50	5.47 ± 0.22	14.85 ± 1.57	2.25 ± 0.34
Average	86.10 ± 19.69	59.13 ± 6.33	34.10 ± 1.89	74.97 ± 2.80	5.46 ± 0.45	15.05 ± 4.43	2.29 ± 0.71
Honeydew	104.09 ± 12.06 *	63.15 ± 5.38 *	35.65 ± 1.12 *	76.10 ± 1.08 *	5.71 ± 0.30 *	18.94 ± 4.36 *	3.18 ± 0.23 *
Blossom	76.51 ± 15.89	56.98 ± 5.77	33.28 ± 1.69	74.36 ± 3.23	5.32 ± 0.47	12.98 ± 2.79	1.82 ± 0.30

*—Significant different between honeydew honey and blossom honey at *p* < 0.05.

**Table 7:** Factor loadings for parameters of stingless bee honey samples.

	Data Related to Figure 1a		Data Related to Figure 1b	
Principal Component (PC) Number	1	2	1	2
Eigenvalue	11.693	5.220	6.952	0.666
% variance	39.00%	17.40%	77.20%	7.40%
Component score correlation				
Color intensity	−0.634	−0.698		
Moisture content	−0.764	−0.407		
Water activity	−0.560	−0.390		
Total soluble solid	0.763	0.408		
Proline	0.285	−0.608		
Electrical conductivity	−0.465	0.720		
Ash content	−0.822	0.334	−0.891	0.310
Diastase	−0.330	0.206		
Hydrogen peroxide	−0.886	−0.147	−0.878	−0.324
pH	−0.107	0.179		
Free acidity	−0.842	−0.201	−0.811	−0.438
Gluconic acid	0.288	0.686		
Acetic acid	0.164	0.715		
HMF	−0.040	−0.666		
Na	−0.710	0.268		
K	−0.817	0.235	−0.892	0.307
Mg	−0.876	0.245	−0.876	0.158
Ca	−0.863	−0.127	−0.810	−0.229
Fe	−0.452	0.413		
Zn	−0.375	0.654		
Mn	−0.137	0.450		
Cr	0.159	0.005		
Total mineral elements	−0.916	0.250	−0.945	0.219
Total phenolic compounds	−0.817	−0.285	−0.851	−0.204
ABTS radical scavenging activity	−0.653	−0.196		
DPPH radical scavenging activity	−0.300	0.478		
Superoxide radical scavenging activity	−0.632	−0.259		
Peroxyl radical inhibition	−0.598	−0.302		
Iron chelation	−0.658	0.290		
Ferric reducing power	−0.907	0.178	−0.945	0.110

**Table 8:** Details of stingless bee honey samples.

Sample	Bee Species	Nectar Source	Botanical Origin	Harvest Time
S1	*Heterotrigona itama*	Acacia tree (*Acacia mangium*)	Honeydew	August 2016
S2	*Heterotrigona itama*	Acacia tree (*Acacia mangium*)	Honeydew	November 2016
S3	* Heterotrigona itama*	Acacia tree (*Acacia mangium*)	Honeydew	April 2017
S4	*Heterotrigona itama*	Acacia tree (*Acacia mangium*)	Honeydew	July 2017
S5	*Heterotrigona itama*	Acacia tree (*Acacia mangium*)	Honeydew	September 2017
S6	*Heterotrigona itama*	Acacia tree (*Acacia mangium*)	Honeydew	April 2018
S7	*Heterotrigona itama*	Acacia tree (*Acacia mangium*)	Honeydew	July 2018
S8	*Heterotrigona itama*	Acacia tree (*Acacia mangium*)	Honeydew	September 2018
S9	*Heterotrigona itama*	Multifloral	Blossom	August 2016
S10	*Heterotrigona itama*	Multifloral	Blossom	November 2016
S11	*Heterotrigona itama*	Multifloral	Blossom	May 2017
S12	*Heterotrigona itama*	Multifloral	Blossom	July 2017
S13	*Heterotrigona itama*	Multifloral	Blossom	September 2017
S14	*Heterotrigona itama*	Multifloral	Blossom	April 2018
S15	*Heterotrigona itama*	Multifloral	Blossom	May 2018
S16	*Heterotrigona itama*	Multifloral	Blossom	July 2018
S17	*Geniotrigona thoracica*	Multifloral	Blossom	October 2016
S18	*Geniotrigona thoracica*	Multifloral	Blossom	December 2016
S19	*Geniotrigona thoracica*	Multifloral	Blossom	April 2017
S20	*Geniotrigona thoracica*	Multifloral	Blossom	July 2017
S21	*Geniotrigona thoracica*	Multifloral	Blossom	March 2018
S22	*Geniotrigona thoracica*	Multifloral	Blossom	June 2018
S23	*Geniotrigona thoracica*	Multifloral	Blossom	October 2018

## Data Availability

The data presented in this study are available in Supplementary Materials here.

## Supplementary Materials

The following are available. Table S1: ^13^C chemical shifts of sugars in each model compound. Table S2: Assignment of the carbon resonances and comparison of the measured amount (%, g/100 g) to that of the actual weight amount of each tautomer in the ^13^C NMR spectra of isoglucose. Table S3: Assignment of the carbon resonances and comparison of the measured amount (%, g/100 g) to that of the actual weight amount of each tautomer in the ^13^C NMR spectra of artificial honey. Figure S1a: Glucose. Figure S1b: Fructose. Figure S1c: Sucrose. Figure S2: Isoglucose. Figure S3: Artificial honey mixture. Figure S4: ^13^C NMR spectra of stingless bee honey samples originating from (A–B) honeydew and (C–D) blossom. S5a: *Heterotrigona itama*. Figure S5b: *Geniotrigona thoracica*. Figure S6. The location of honey sample collection in peninsular Malaysia.

## Sample Availability

Stingless bee honey samples are available from the authors.
