# Supplementary material for: Botanical Origin Differentiation of Malaysian Stingless Bee Honey Produced by Heterotrigona itama and Geniotrigona thoracica Using Chemometrics
Source: Molecules. 2021 Dec 16;26(24):7628. doi: 10.3390/molecules26247628 (PMC8704392; doi:10.3390/molecules26247628)
Supplement: Supplementary file 1 [file molecules-26-07628-s001.zip › molecules-1494850-supplementary.pdf]

Supplementary Materials

Table S1: ^{13}C chemical shifts of sugars in each model compound.

Sugar	Tautomer %	C1	C2	C3	C4	C5	C6	C1´	C2´	C3´	C4´	C5´	C6´
Glucose													
α-D-glucopyranose (α-GP)	37.23	92.7	72.1	73.4	70.2	72.0	61.2	-	-	-	-	-	-
β-D-glucopyranose (β-GP)	62.77	96.5	74.7	76.4	70.3	76.6	61.4	-	-	-	-	-	-
Fructose													
α-D-fructopyranose (α-FP)	3.64	65.8	-	-	70.6	71.1	61.7	-	-	-	-	-	-
β-D-fructopyranose (β-FP)	69.18	64.5	98.7	68.1	70.3	69.8	64.0	-	-	-	-	-	-
α-D-fructofuranose (α-FF)	5.97	63.5	105.0	82.6	76.6	81.9	61.7	-	-	-	-	-	-
β-D-fructofuranose (β-FF)	21.21	63.2	102.1	76.0	75.1	81.3	63.0	-	-	-	-	-	-
Sucrose	-	92.8	71.7	73.2	69.8	73.0	60.7	61.9	104.3	77.0	74.6	82.0	63.0

Table S2: Assignment of the carbon resonances and comparison of the measured amount (%, g/100 g) to that of the actual weight amount of each tautomer in the ^{13}C NMR spectra of isoglucose.

Sugar	Tautomer %	Chemical shift	Average integration value	Actual concentration	Concentration determined by ^{13}C NMR	Similarity (%)
Glucose						
α-D-glucopyranose (α-GP)	39.17	72.0, 72.1, 73.4, 92.7	0.2350	17.63	18.08	97.45
β-D-glucopyranose (β-GP)	60.83	74.7, 96.5	0.3650	27.37	28.09	97.37
Fructose						
α-D-fructopyranose (α-FP)	3.82	65.8, 70.7, 71.1	0.0268	2.10	2.06	98.10
β-D-fructopyranose (β-FP)	69.13	64.5, 68.2, 69.8, 98.7	0.4850	38.02	37.21	97.87
α-D-fructofuranose (α-FF)	5.20	63.5, 81.9, 82.6, 105.0	0.0345	2.86	2.80	97.90
β-D-fructofuranose (β-FF)	21.85	63.0, 63.3, 75.1, 76.0, 81.3, 102.1	0.1533	12.02	11.76	97.84

Table S3: Assignment of the carbon resonances and comparison of the measured amount (%, g/100 g) to that of the actual weight amount of each tautomer in the ^{13}C NMR spectra of artificial honey.

**Sugar**	**Tautomer %**	**Chemical shift**	**Average integration value**	**Actual concentration**	**Concentration determined by ^{13}C NMR**	**Similarity (%)**
Glucose						
α-D-glucopyranose (α-GP)	38.46	72.0, 72.1, 73.4	0.15	15.87	17.02	92.75
β-D-glucopyranose (β-GP)	61.64	96.5	0.24	25.44	27.28	92.77
Fructose						
α-D-fructopyranose (α-FP)	2.51	65.8	0.0106	1.27	1.21	95.28
β-D-fructopyranose (β-FP)	70.87	64.5, 68.2, 98.7	0.3000	35.99	34.05	94.61
α-D-fructofuranose (α-FF)	5.32	63.5, 82.6, 105.0	0.0225	2.70	2.56	94.81
β-D-fructofuranose (β-FF)	21.31	63.3, 75.1, 76.0, 81.3, 102.1	0.0902	10.82	10.24	94.64
Sucrose	-	60.7, 71.6, 73.0, 73.2, 77.0, 104.3	0.0357	7.94	7.69	96.85

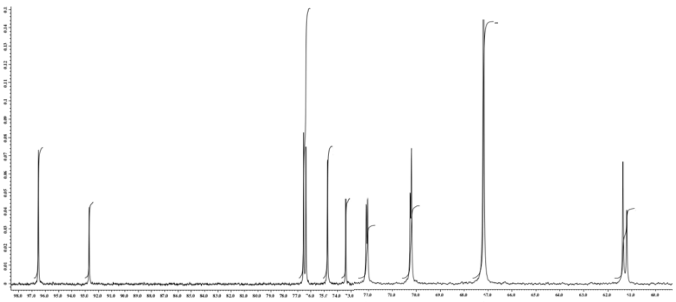

Figure S1a: Glucose.

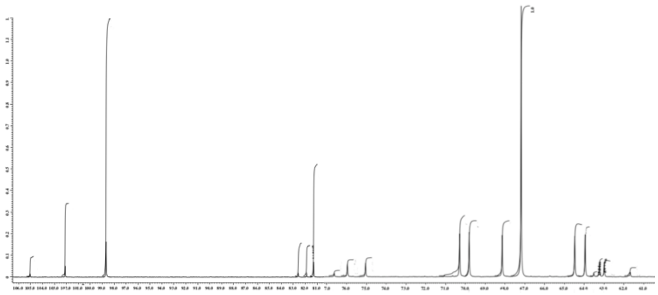

Figure S1b: Fructose.

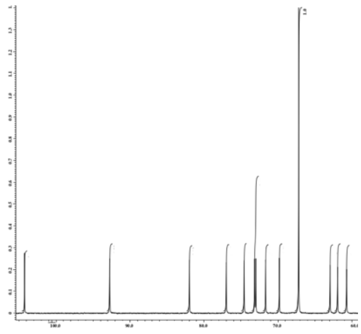

Figure S1c: Sucrose.

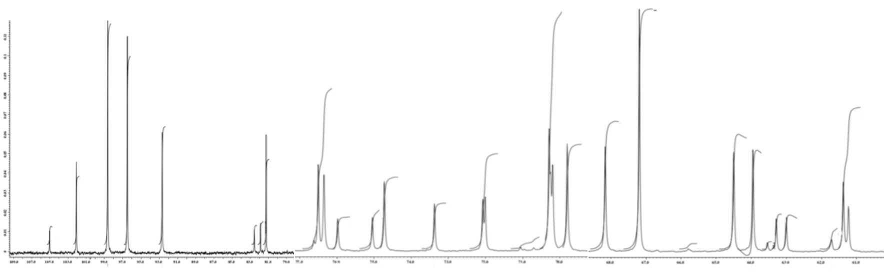

Figure S2: Isoglucose.

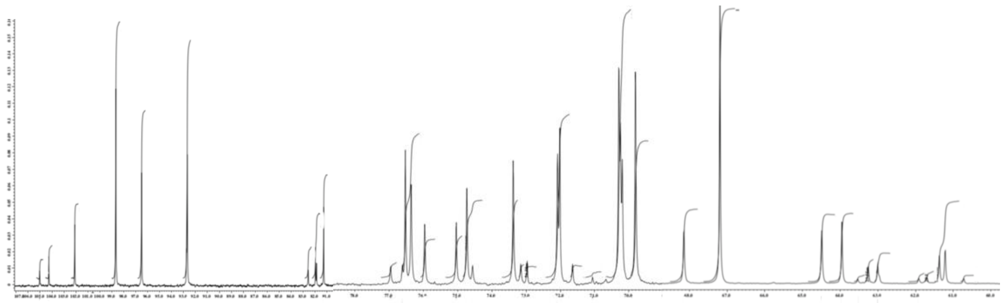

Figure S3: Artificial honey mixture.

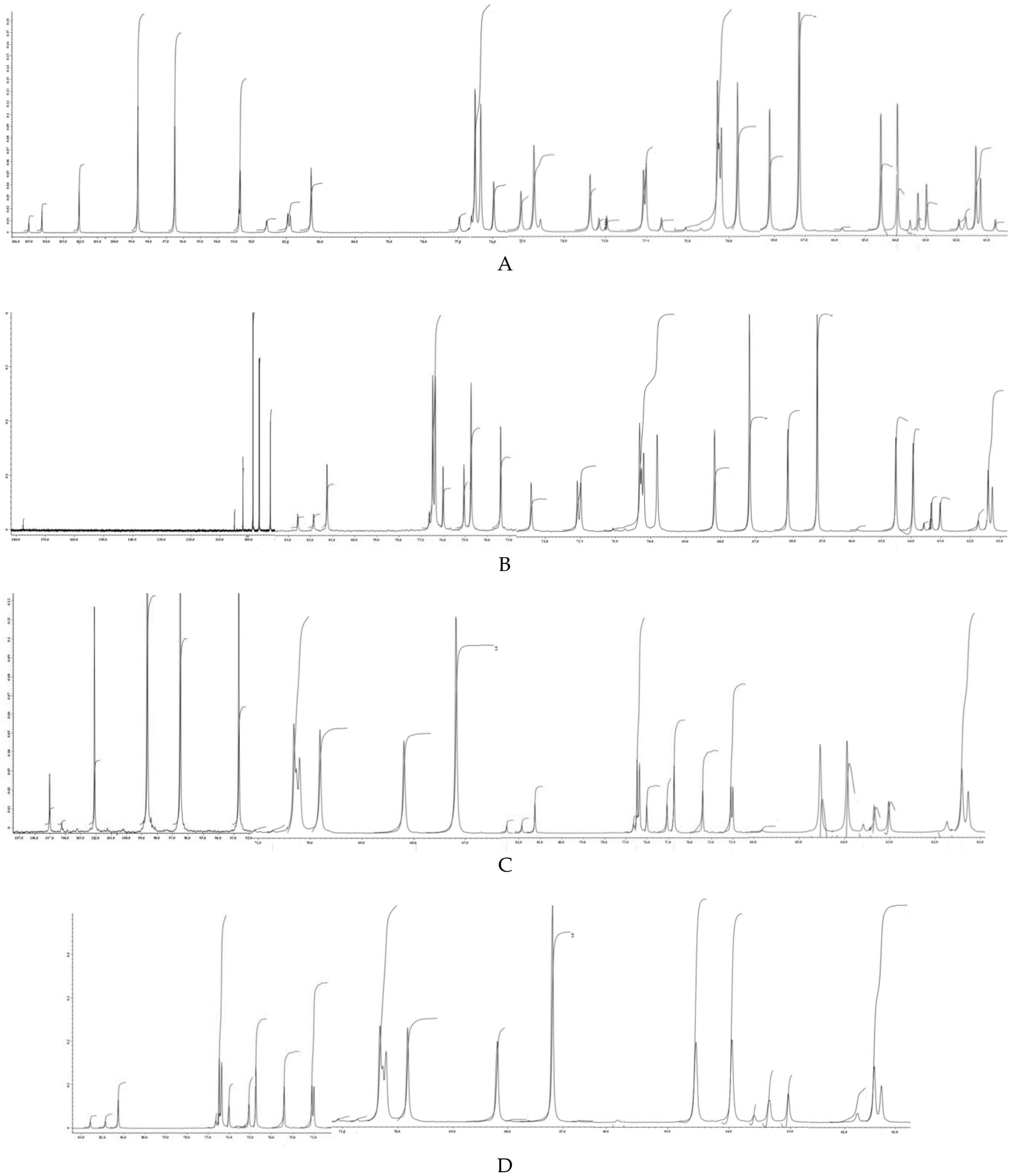

Figure S4: ^{13}C NMR spectra of stingless bee honey samples originating from (A-B) honeydew and (C-D) blossom.

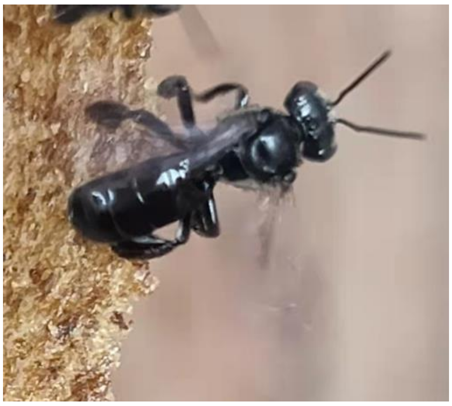

Figure S5a: *Heterotrigona itama*.

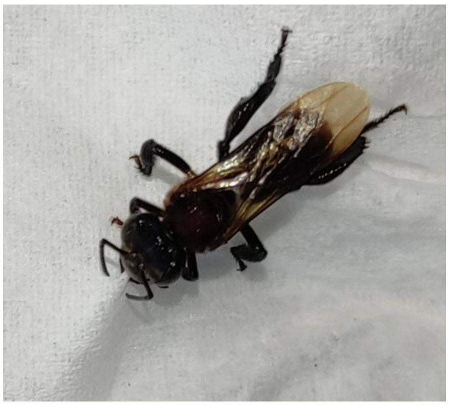

Figure S5b: *Geniotrigona thoracica*.

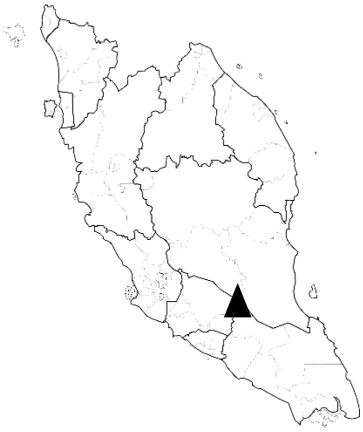

Figure S6. The location of honey sample collection in peninsular Malaysia.
